# Discovery of the Seven-ring Polycyclic Aromatic Hydrocarbon Cyanocoronene (C_24_H_11_CN) in GOTHAM Observations of TMC-1

**DOI:** 10.3847/2041-8213/adc911

**Published:** 2025-04-30

**Authors:** Gabi Wenzel, Siyuan Gong, Ci Xue, P. Bryan Changala, Martin S. Holdren, Thomas H. Speak, D. Archie Stewart, Zachary T. P. Fried, Reace H. J. Willis, Edwin A. Bergin, Andrew M. Burkhardt, Alex N. Byrne, Steven B. Charnley, Andrew Lipnicky, Ryan A. Loomis, Christopher N. Shingledecker, Ilsa R. Cooke, Michael C. McCarthy, Anthony J. Remijan, Alison E. Wendlandt, Brett A. McGuire

**Affiliations:** 1 Department of Chemistry, Massachusetts Institute of Technology, Cambridge, MA 02139, USA; gwenzel@mit.edu, brettmc@mit.edu; 2 Center for Astrophysics ∣ Harvard & Smithsonian, Cambridge, MA 02138, USA; 3 JILA, University of Colorado Boulder and National Institute of Standards and Technology, Boulder, CO 80309, USA; 4 Department of Physics, University of Colorado Boulder, Boulder, CO 80309, USA; 5 Department of Chemistry, University of British Columbia, Vancouver, BC, Canada; 6 Department of Astronomy, University of Michigan, Ann Arbor, MI 48109, USA; 7 Department of Earth, Environment, and Physics, Worcester State University, Worcester, MA 01602, USA; 8 Astrochemistry Laboratory and the Goddard Center of Astrobiology, Solar System Exploration Division, NASA Goddard Space Flight Center, Greenbelt, MD 20771, USA; 9 National Radio Astronomy Observatory, Charlottesville, VA 22903, USA; 10 Department of Chemistry, Virginia Military Institute, Lexington, VA 24450, USA

## Abstract

We present the synthesis and laboratory rotational spectroscopy of the seven-ring polycyclic aromatic hydrocarbon (PAH) cyanocoronene (C_24_H_11_CN) using a laser-ablation-assisted cavity-enhanced Fourier transform microwave spectrometer. A total of 71 transitions were measured and assigned between 6.8 and 10.6 GHz. Using these assignments, we searched for emission from cyanocoronene in the Green Bank Telescope (GBT) Observations of TMC-1: Hunting Aromatic Molecules project observations of the cold dark molecular cloud TMC-1 using the 100 m GBT. We detect a number of individually resolved transitions in ultrasensitive *X*-band observations and perform a Markov Chain Monte Carlo analysis to derive best-fit parameters, including a total column density of $N({{\mathrm{C}}}_{24}{{\mathrm{H}}}_{11}{\mathrm{CN}})=2.6{9}_{-0.23}^{+0.26}\times 1{0}^{12}\,{{\mathrm{cm}}}^{-2}$ at a temperature of $6.0{5}_{-0.37}^{+0.38}\,$K. A spectral stacking and matched filtering analysis provides a robust 17.3*σ* significance to the overall detection. The derived column density is comparable to that of cyano-substituted naphthalene, acenaphthylene, and pyrene, defying the trend of decreasing abundance with increasing molecular size and complexity found for carbon chains. We discuss the implications of the detection for our understanding of interstellar PAH chemistry and highlight major open questions and next steps.

## 1. Introduction

Polycyclic aromatic hydrocarbons (PAHs) are a class of molecules thought to sequester a substantial portion (10%–25%) of the interstellar carbon budget (E. Dwek et al. 1997; E. Habart et al. 2004; A. G. G. M. Tielens 2008; M. Chabot et al. 2020); contribute to interstellar H_2_ formation both as catalytic surfaces (E. Rauls & L. Hornekær 2008; V. Le Page et al. 2009; V. Mennella et al. 2012; J. D. Thrower et al. 2012); through H abstraction (C. W. Bauschlicher 1998; L. Boschman et al. 2015), serve as charge-balance carriers (E. L. O. Bakes & A. G. G. M. Tielens 1998; F. Carelli & F. A. Gianturco 2012), and contribute to neutral gas heating in interstellar sources (O. Berné et al. 2022), among numerous other roles. Since the mid-1980s, the unidentified infrared bands (UIRs), broad-emission features in the mid-infrared range observed toward many astronomical objects that are classified as photon-dominated regions (PDRs) of the interstellar medium (ISM), have been widely attributed to vibrational modes of electronically excited PAHs (A. Léger & J. L. Puget 1984; L. J. Allamandola et al. 1985)—for this reason, these bands are also commonly referred to as the aromatic infrared bands (AIBs)—although no individual PAH carrier has been identified via its infrared emission (A. G. G. M. Tielens 2008). Definitive evidence for the presence of PAHs outside the solar system, however, was obtained in 2021 with the discoveries of 1- and 2-cyanonaphthalene (C_10_H_7_CN) via radio astronomy (B. A. McGuire et al. 2021). The detections were made in the cold, starless molecular cloud TMC-1 with the 100 m Robert C. Byrd Green Bank Telescope (GBT) as part of the GBT Observations of TMC-1: Hunting Aromatic Molecules (GOTHAM) large program. Shortly thereafter, a third PAH, indene (C_9_H_8_; A. M. Burkhardt et al. 2021; J. Cernicharo et al. 2021a), was discovered both by GOTHAM and the Yebes 40 m telescope *Q*-band Ultrasensitive Inspection Journey to the Obscure TMC-1 Environment (QUIJOTE) project, followed the next year with the detection of 2-cyanoindene (C_9_H_7_CN; M. L. Sita et al. 2022) by GOTHAM. The detections of 1- and 5-cyanoacenaphthylene (C_12_H_7_CN) were then reported by J. Cernicharo et al. (2024).

Most recently, 1-, 2-, and 4-cyanopyrene (C_16_H_9_CN) were discovered in the GOTHAM observations (G. Wenzel et al. 2024, 2025). These four-ring, pericondensed PAHs were the largest species detected with radio astronomy to date. From these observations, it was estimated that the unsubstituted (bare) pyrene (C_16_H_10_) itself may account for as much as 0.1% of the carbon budget (G. Wenzel et al. 2024). Notably, the derived column densities of the known PAHs in TMC-1 are all similar: the cyanonaphthalenes, cyanoacenaphthylenes, and cyanopyrenes all lie within a factor of ∼2 of each other between 0.75–1.5 × 10^12^ cm^−2^. (2-cyanoindene is somewhat lower at 0.2 × 10^12^ cm^−2^.) This is in stark contrast to the generally observed column density trends with carbon atom numbers within a molecular family (R. A. Loomis et al. 2021; M. A. Siebert et al. 2022). Given these results, we considered whether yet larger PAHs may be detectable with a similar column density in TMC-1.

Coronene (C_24_H_12_) is often described in the literature as the “prototypical” pericondensed (compact) PAH, renowned for its structural, chemical, and spectral properties, making it a model for understanding larger PAHs in terrestrial and extraterrestrial environments. Its *D*_6*h*_ symmetry and highly conjugated *π*-electron system enhance its aromatic character and stability, making it energetically favorable and distinguishing it from simpler PAHs like naphthalene and anthracene (E. Clar 1964, 1983). Indeed, the compellingly close agreement with small shifts (≤0.6 *μ*m) between the computed vibrational features of coronene and the observed AIBs was used by A. Léger & J. L. Puget (1984) in their original suggestion that PAHs were the primary carriers. Due to its role as a “model PAH,” many astrochemical studies have focused on coronene and its derivatives, clusters, fragments, and charge states. Coronene is efficiently produced at high temperatures due to its thermodynamic stability (S. Stein 1978; D. M. Hudgins & L. J. Allamandola 1995), is the largest PAH found in the Murchison meteorite (M. A. Sephton et al. 2004; H. Sabbah et al. 2017), and is among the PAHs identified in return samples from asteroid Ryugu (H. Sabbah et al. 2024). Its infrared spectrum is known for its neutral form at varying temperatures (C. Joblin et al. 1995) and for its cationic form in ion trap experiments (J. Oomens et al. 2001). The infrared spectrum of protonated coronene (C_24_H_13_^+^) reveals it to be a key species among the potential carriers of UIR bands (or AIBs; O. Dopfer 2011; M. Bahou et al. 2014; S. Cazaux et al. 2016). The destruction of coronene may produce key reactive intermediates involved in the formation of smaller complex organic molecules and fullerenes (T. Chen et al. 2020; M. Gatchell et al. 2021; S. Panchagnula et al. 2024). However, no acetylene loss, one of the major fragmentation channels of smaller PAHs, has been observed for coronene cations in experiments resembling PDRs with vacuum ultraviolet photons up to 20 eV (J. Zhen et al. 2016; C. Joblin et al. 2020), further emphasizing its extreme stability. Coronene’s role in the context of the diffuse interstellar bands (DIBs) has been debated. Dehydrogenated, protonated, and cationic coronene were proposed as potential carriers of DIBs (G. Malloci et al. 2008; A. Pathak & P. J. Sarre 2008), but these hypotheses were disproven (F. Useli-Bacchitta et al. 2010; I. Garkusha et al. 2011; F.-X. Hardy et al. 2017). Nevertheless, some distribution of PAHs remains promising DIB carrier candidates due to their close relation to the fullerene family (E. K. Campbell et al. 2015).

As with many PAHs, coronene possesses no permanent electric dipole moment and thus no pure-rotational spectrum. A search for its CN-substituted variant is therefore reasonable, and indeed, experimental evidence has previously shown that the addition of CN to coronene may proceed readily under interstellar conditions (M. P. Bernstein et al. 2002) although we note that the study in question was specifically in the condensed phase. Here, we present a combined computational and laboratory study of the rotational spectrum of cyanocoronene (C_24_H_11_CN) and its subsequent discovery in TMC-1.

## 2. Synthesis

**Figure 1.**
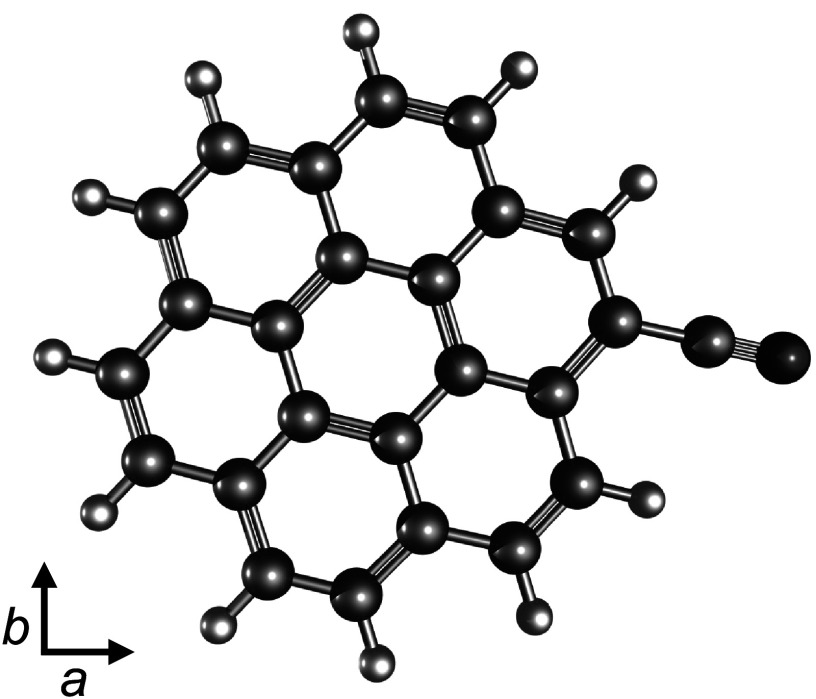
Optimized geometry of cyanocoronene, C_24_H_11_CN, in its principal axis system spanned by the vectors *a* and *b*. The highly symmetric coronene has 12 identical carbon sites for CN substitution that all result in the same cyanocoronene. Its permanent electric dipole moment was calculated to be *μ*_*a*_ = 5.67 D and *μ*_*b*_ = 0.59 D.

Cyanocoronene, the CN derivative of the highly symmetric PAH coronene (see Figure 1), is not commercially available. To measure the laboratory rotational spectrum of cyanocoronene, we synthesized it via the route described in detail in Appendix A, which was partially based on previous work (T. J. Dale & J. Rebek 2006; K. Hyodo et al. 2017). Briefly, coronene (C_24_H_12_) was purchased from Ambeed Inc. (purity ∼98%) and used as the starting material to prepare formylcoronene (C_24_H_11_CHO). Formylcoronene further reacted via formylcoronene-oxime (C_24_H_11_CHNOH) to cyanocoronene (C_24_H_11_CN), a yellow solid, with an approximate yield of 59%.

## 3. Laboratory Spectroscopy

The geometry of cyanocoronene (see Figure 1) was first optimized at the *ω*B97X-D4/def2-TZVPP level (F. Weigend 2006; J.-D. Chai & M. Head-Gordon 2008; E. Caldeweyher et al. 2017) in ORCA 5.0.4 (F. Neese 2022) and re-optimized at the B3LYP/aug-cc-pVTZ level (T. H. Dunning 1989; R. A. Kendall et al. 1992; A. D. Becke 1993; E. R. Davidson 1996) in Gaussian 16 (M. J. Frisch et al. 2016), which has recently been shown to perform well when obtaining rotational constants of PAHs (E. M. Neeman & A. Lesarri 2025). We further benchmarked its performance using the three cyanopyrene isomers previously studied (see Appendix B and Table B1). Together with quartic centrifugal distortion constants derived from the harmonic force field (see Table 1), these theoretically calculated spectroscopic constants formed the basis of our laboratory search.

**Table 1:** Rotational Constants of Cyanocoronene in the A-reduced III^*l*^ Representation

Parameter	B3LYP	Experimental^a^
	aug-cc-pVTZ	
*A* (MHz)	335.393	333.852989(249)
*B* (MHz)	222.035	221.2700880(838)
*C* (MHz)	133.594	133.1081015(122)
Δ_*J*_ (Hz)	0.669	[0.669]
Δ_*JK*_ (Hz)	−0.617	[−0.617]
Δ_*K*_ (Hz)	0.011	[0.011]
*δ*_*J*_ (Hz)	−0.120	[−0.120]
*δ*_*K*_ (Hz)	−0.494	[−0.494]
${N}_{{\mathrm{lines}}}^{{\mathrm{fit}}}$	⋯	71
${N}_{{\mathrm{lines}}}^{{\mathrm{unique}}}$	⋯	38
*σ*_fit_ (kHz)	⋯	2.595
${(J,{K}_{a})}_{{\mathrm{\max }}}$	⋯	(39, 10)

**Note.** ${N}_{{\mathrm{lines}}}^{{\mathrm{fit}}}$ and ${N}_{{\mathrm{lines}}}^{{\mathrm{unique}}}$ refer to the number of distinct transitions in the fit and number of unique transition frequencies in the fit, respectively. See Table C1 for the line list.

^a^ Values in parentheses are 1​​​​​​*σ* uncertainties in units of the last digit. Values in brackets were not determinable and fixed to the theoretically calculated constants.

Details of the sample preparation and spectroscopic measurements are given in G. Wenzel et al. (2024). Briefly, a hydraulic press was used to compress approximately 600 mg of the solid sample into a cylindrical sample rod, which was then mounted in a rotating stage located downstream of a pulsed valve backed with neon carrier gas. The rod was then ablated using the second harmonic at 532 nm of a Nd:YAG laser to inject the sample into the gas phase, where it was entrained in the neon and supersonically expanded into the cavity of a Balle–Flygare-type Fourier transform microwave (FTMW) spectrometer (T. J. Balle & W. H. Flygare 1981; K. N. Crabtree et al. 2016). At the ∼2 K rotational temperatures generated by the supersonic expansion, the strongest transitions of cyanocoronene fall in the lowest end of the operational range of the instrument, between 6 and 8 GHz.

In total, we were able to measure and assign 71 transitions (38 unique lines) of cyanocoronene (see Table C1). We used SPCAT/SPFIT (H. M. Pickett 1991) to determine the spectroscopic constants of cyanocoronene by least-squares fitting in a Watson A reduction (III^*l*^ representation). The asymmetry parameter, *κ* = −0.12, places cyanocoronene far from either the prolate or oblate symmetric-top limits. Centrifugal distortion constants were fixed to the theoretically calculated values. The ^14^N nuclear electric quadrupole coupling was neglected entirely because no splittings could be resolved for the high-*J* transitions measured (see Table C1). The best-fit rotational constants, *A*, *B*, and *C*, are listed in Table 1 and with a mean absolute percentage error (MAPE) of 0.39%, in excellent agreement with the calculated values.

## 4. Observations

**Figure 2.**
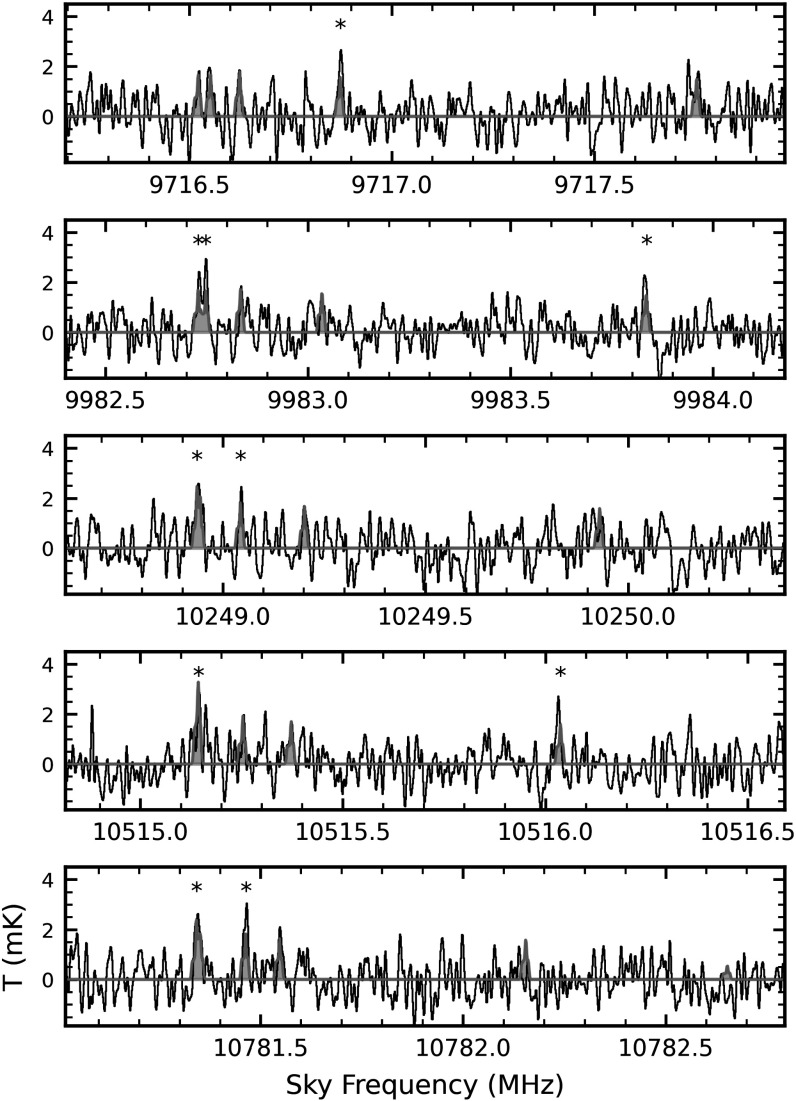
GOTHAM spectra smoothed with a 10-channel Hanning window to a resolution of 14 kHz (black) overlaid with the spectra of cyanocoronene (orange) simulated using the MCMC-derived parameters given in Table E2. Lines with SNR  > 3*σ* are marked with asterisks.

The observations of TMC-1 were acquired on the 100 m Robert C. Byrd GBT as part of the GOTHAM project (B. A. McGuire et al. 2020). Details of the observing strategy and data reduction procedures are provided elsewhere (B. A. McGuire et al. 2020, 2021; M. L. Sita et al. 2022). For this analysis, we used observations from the fifth data reduction, which includes advances in artifact and radio-frequency interference removal, atmospheric opacity corrections, and baseline removal (C. Xue 2024). Briefly, we target the “cyanopolyyne peak” of TMC-1 centered at *α*_J2000_ = 04$\mathop{.}\limits^{{\mathrm{h}}}$41$\mathop{{\mathrm{.}}}\limits^{{\mathrm{m}}}$42.5$\mathop{{\mathrm{.}}}\limits^{{\mathrm{s}}}$, *δ*_J2000_ = +25°41$^{\prime} $26$\mathop{.}\limits^{\unicode{x02033}}$8. The GOTHAM observations cover 29 GHz of bandwidth between 4 and 36 GHz, limited by gaps in receiver coverage, at a resolution of 1.4 kHz with a rms noise level between 3.8 and 15.0 mK across most of the observed frequency range. Flux calibration was achieved with switched noise-diode measurements, resulting in an estimated antenna temperature accuracy of 10%–20%. In the case of cyanocoronene, the strongest transitions contributing to the overall detection fall in the *C*, *X*, and *Ku*-bands. As discussed below, the observation of individual transitions of cyanocoronene was enabled by our ultrasensitive observations in *X*-band between 9.38 and 10.96 GHz (GBT Project 24A-124). Combining this deep *X*-band and previous GOTHAM observations, we reach an rms of 1.5 mK in this frequency range. While we conduct the analysis described in Section 5 on the full-resolution spectra, we also smoothed the data with a 10-channel Hanning window to a resolution of 14 kHz to increase the signal-to-noise ratio (SNR) on individual transitions and better visualize the detection (see Figure 2).

## 5. Astronomical Analysis

Due to the low rms noise in our *X*-band data, the brightest SNR lines of cyanocoronene at TMC-1 conditions (*v*_LSR_ ∼ 5.8 km s^−1^, *T*_ex_ ∼ 5–7 K) fall in the frequency range of 8–12 GHz. A search for cyanocoronene in the GOTHAM data yielded several individually resolved rotational transitions close to the rms noise, particularly near 10.781 GHz (see Figure 2). We performed a Markov Chain Monte Carlo (MCMC) analysis adopted from previous work (R. A. Loomis et al. 2021; B. A. McGuire et al. 2021) to derive physical parameters that best describe the emission of cyanocoronene. This approach provides an inference by conditioning the data on priors (see Table E1) and sampling posterior distributions. The uncertainty of observations is computed as the quadrature sum of the local rms noise and an additional 20% systematic uncertainty. The parameters derived from the MCMC analysis are listed in Table E2, where the velocities in the local standard of rest, *v*_LSR_, and excitation temperature, *T*_ex_, are consistent with prior detections in TMC-1 (M. C. McCarthy et al. 2021; M. L. Sita et al. 2022). The derived uncertainties in each parameter reflect the posterior probability distribution.

**Figure 3.**
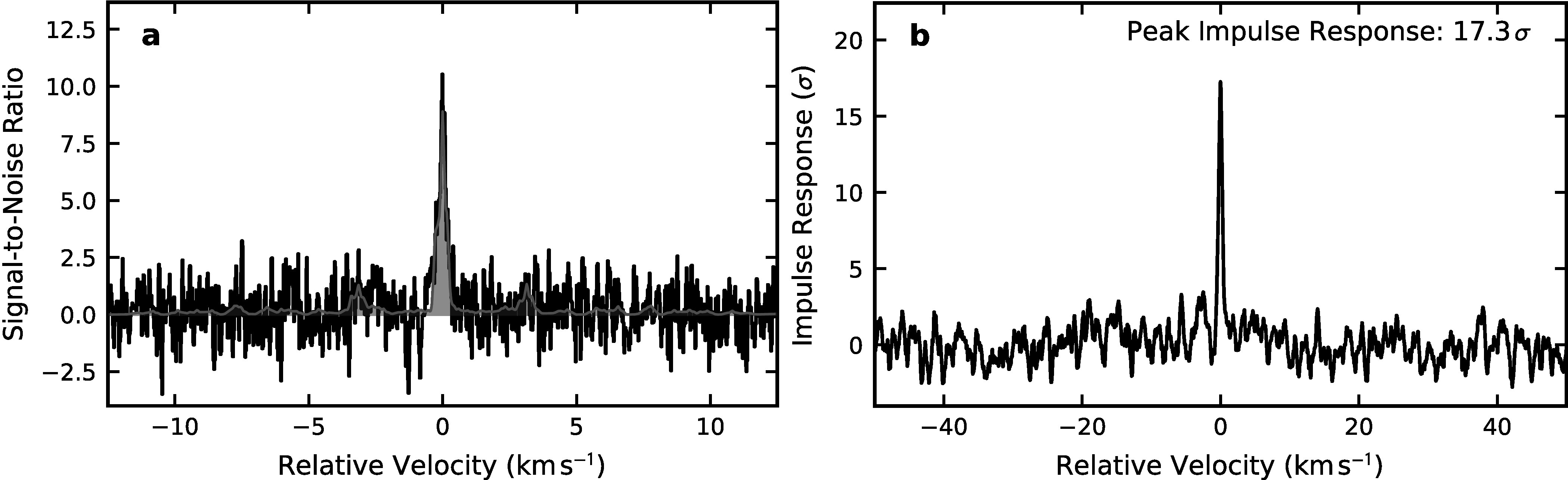
(a) Velocity-stacked spectra and (b) matched-filter analysis results of cyanocoronene spectra in the GOTHAM data generated using the methodologies outlined in R. A. Loomis et al. (2021).

From our MCMC analysis, the total column density was derived as the sum of the column densities of all four Doppler components, yielding a value of $N({{\mathrm{C}}}_{24}{{\mathrm{H}}}_{11}{\mathrm{CN}})$ = $2.6{9}_{-0.23}^{+0.26}\times 1{0}^{12}\,{{\mathrm{cm}}}^{-2}$ and a ${T}_{{\mathrm{ex}}}=6.0{5}_{-0.37}^{+0.38}\,$K. This value of *T*_ex_ is consistent with other PAHs detected in TMC-1 (B. A. McGuire et al. 2021; J. Cernicharo et al. 2024; G. Wenzel et al. 2024, 2025). To quantify the significance of our cyanocoronene detection, we performed a velocity-stack and matched-filtering analysis (as described in R. A. Loomis et al. 2021; B. A. McGuire et al. 2021; G. Wenzel et al. 2024) of the 100 brightest SNR lines of cyanocoronene, resulting in a positive detection with a high confidence level of 17.3*σ* in the matched-filter response (see Figure 3).

## 6. Discussion

**Figure 4.**
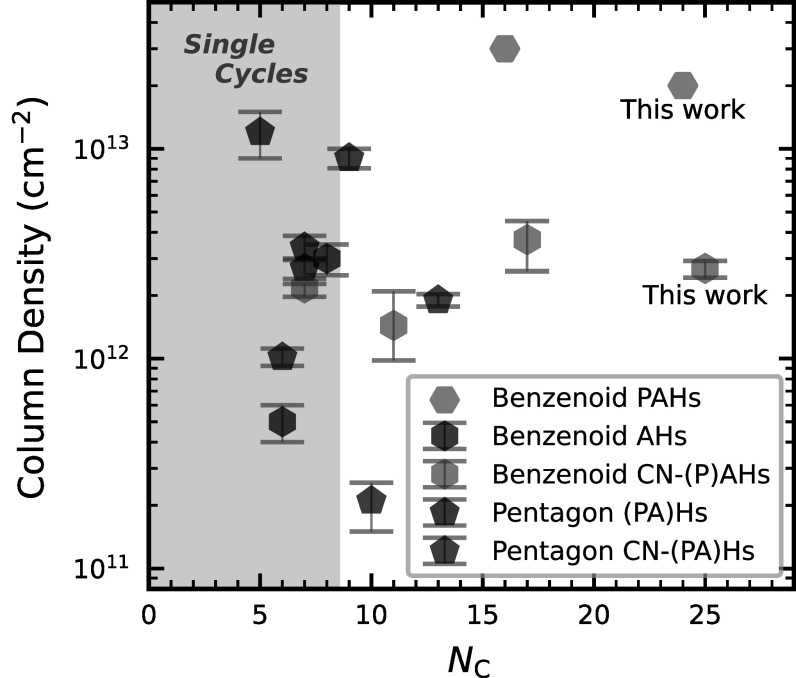
Comparison of the derived column densities of the cyclic hydrocarbon (H) population in TMC-1 vs. number of carbon atoms, *N*_C_. We distinguish between single and multiple cycles, pentagon-containing (PA)Hs, solely hexagon-containing (P)AHs, and their CN derivatives. Purple hexagons refer to pure benzenoid PAHs, and their column densities were estimated from their CN derivatives (see text and Appendix G for details). Black hexagons are pure, single benzenoid cycles (aromatic hydrocarbons, AHs). Light blue hexagons correspond to CN-substituted benzenoid (P)AHs; black and dark blue pentagons are pure and CN-substituted pentagonal (PA)Hs, respectively. Column density values and the references they were taken from are reported in Table F1.

**Figure 5.**
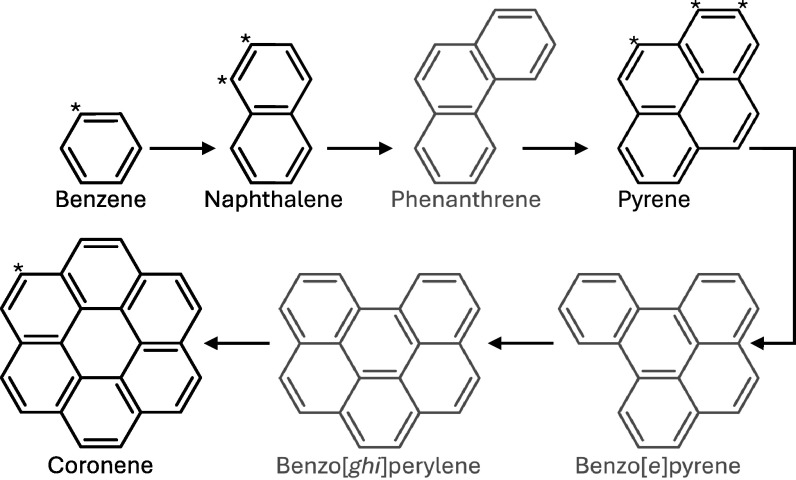
First seven members of the most thermodynamically favorable, high-temperature (P)AH polymerization route, adapted from S. Stein (1978) and D. M. Hudgins & L. J. Allamandola (1995). Note that this is not a reaction scheme. Those whose CN derivatives have been detected in TMC-1 are depicted in black with their substitution sites marked by asterisks. Those in gray have not (yet) been detected or searched for in TMC-1. See Section 6 for details.

Based on the assumption that CN addition to aromatic double bonds is barrierless (N. Balucani et al. 2000; D. E. Woon & E. Herbst 2009; I. R. Cooke et al. 2020), we use the detected CN derivatives as proxies to infer the presence of their unsubstituted parent PAHs in TMC-1. Analogous to our estimate of the column density of pyrene in TMC-1 (G. Wenzel et al. 2025), we use the CN/H ratio to derive an approximate column density of coronene of *N*(C_24_H_12_) ≈ 2 × 10^13^ cm^−2^ (see Appendix G for details). Cyanocoronene, with its 24 carbon atoms (excluding the CN group), is by far the largest PAH found to date by radio telescopes in the ISM. Together with the previous detections of mono- and polycyclic (aromatic) hydrocarbons in TMC-1, which have an approximately flat distribution in column density with increasing size (see Figure 4), this result challenges our understanding of the chemistry at play in the dense ISM. Although no low-temperature bottom-up gas-phase formation routes are known for compact medium-sized PAHs such as pyrene, it is remarkable that the detected CN derivatives of the mono- and polycyclic aromatic hydrocarbons benzene, naphthalene, pyrene, and now coronene, are all members of the most thermodynamically stable PAHs formed by the high-temperature polymerization route (see Figure 5; S. Stein 1978; D. M. Hudgins & L. J. Allamandola 1995). This polymerization route builds up large, highly pericondensed PAHs with low H/C ratios by adding one ring at a time, e.g., via the H-abstraction acetylene (C_2_H_2_) addition (HACA) mechanism (E. Reizer et al. 2022).

Considering environments of circumstellar envelopes, L. Zhao et al. (2018) proposed a high-temperature gas-phase formation route for pyrene, starting from 4-phenanthrenyl radical. Indeed, phenanthrene is also a member of the most thermodynamically stable PAH polymerization route (see Figure 5). However, cursory searches for its CN derivative 9-cyanophenanthrene, whose rotational spectrum is known (D. McNaughton et al. 2018), in our GOTHAM observations have not yet been successful. This could be due to the fact that the spectroscopy of only one of the five possible cyanophenanthrene isomers is known, while others might be more abundant. However, it is consistent with the findings by S. S. Zeichner et al. (2023) that the three-ring (linear or noncompact) PAH species identified in return samples from asteroid Ryugu were formed at high temperatures, while two- and four-ring PAH species were formed via a kinetically controlled route at low temperatures (∼10 K), in line with our findings in TMC-1. To our knowledge, neither the other two members of the series, benzo[*e*]pyrene and benzo[*ghi*]perylene, nor their CN-substituted derivatives (six possible individual addition sites per species) have yet been characterized by laboratory rotational spectroscopy. Measuring their laboratory spectra will be crucial for searching for these species in TMC-1 and related sources, especially because high-temperature gas-phase formation routes from benzo[*e*]pyrene and benzo[*ghi*]perylene to coronene in circumstellar envelopes have recently been revealed (S. J. Goettl et al. 2023). Detections of, or upper limit constraints on, such intermediates on the path to larger PAH formation in TMC-1 will be critical for elucidating this new and unexplored cold complex chemistry.

In addition to considering formation routes, however, it is becoming increasingly clear that resilience to the major destruction routes of interstellar molecules likely represents another factor which may help to explain the surprisingly flat abundance of PAHs even out to the size of cyanocoronene. For gas-phase species in molecular clouds, generally, two of the main destruction routes are reactions with ions and depletion onto grains – both of which have been the subject of very recent studies involving PAHs. For ion-neutral reactions, recent ion-beam storage ring experiments by M. H. Stockett et al. (2025) and J. N. Bull et al. (2025) indicate that the underlying PAH backbone can be maintained via efficient radiative cooling, thereby opening up the possibility of a kind of chemical “recycling” of PAHs that would certainly contribute to the high observed abundance of, e.g., cyanocoronene. Turning to depletion onto grains, E. Dartois et al. (2022) conducted experiments on the sputtering yield of solid-phase perylene and coronene bombarded by energetic ions, analogous to the cosmic ray exposure of species in dust-grain ice mantles. They found that such cosmic ray-induced sputtering is efficient under ISM conditions, and even predict a gas-phase fractional abundance of coronene over 10^−10^, in agreement with the findings presented here. The incorporation of such findings into astrochemical models therefore represents a very promising means of substantially improving their agreement with astronomical observations.

## 7. Conclusions

We report the interstellar identification of cyanocoronene, a nitrile derivative of the seven-ring PAH coronene, in GOTHAM observations of the cold molecular cloud TMC-1. We derive high column densities of cyanocoronene and its unsubstituted parent, coronene, of $N({{\mathrm{C}}}_{24}{{\mathrm{H}}}_{11}{\mathrm{CN}})=2.6{9}_{-0.23}^{+0.26}\times 1{0}^{12}\,{{\mathrm{cm}}}^{-2}$ and *N*(C_24_H_12_) ≈ 2 × 10^13^ cm^−2^, respectively. Cyanocoronene is the largest individual PAH discovered in space and is present in similar column density to the four-ring PAH cyanopyrene, suggesting an unexplored reservoir of larger PAHs in the ISM. This discovery delivers additional support for the PAH hypothesis and further evidence of the ubiquitous presence of PAHs in space. Comparisons to organics in the Murchison meteorite and asteroid Ryugu suggest a substantial inheritance of PAHs, possibly produced in the cold (*T* ∼ 10 K) conditions that occur ∼1 Myr before star birth. They represent a promising source of carbon for forming terrestrial worlds in stellar systems, to which carbon is supplied in the form of solid-state organics (J. Li et al. 2021) from their own natal clouds.

## 8. Data Access and Code

The raw data of the GOTHAM observations are publicly available in the GBT Legacy Data Archive.^11^ ^11^
https://greenbankobservatory.org/portal/gbt/gbt-legacy-archive/gotham-data/
 The code used to perform the analysis is part of the molsim open-source package; an archival version of the code can be accessed at B. A. McGuire et al. (2024).

## Figures and Tables

**Figure A1.**
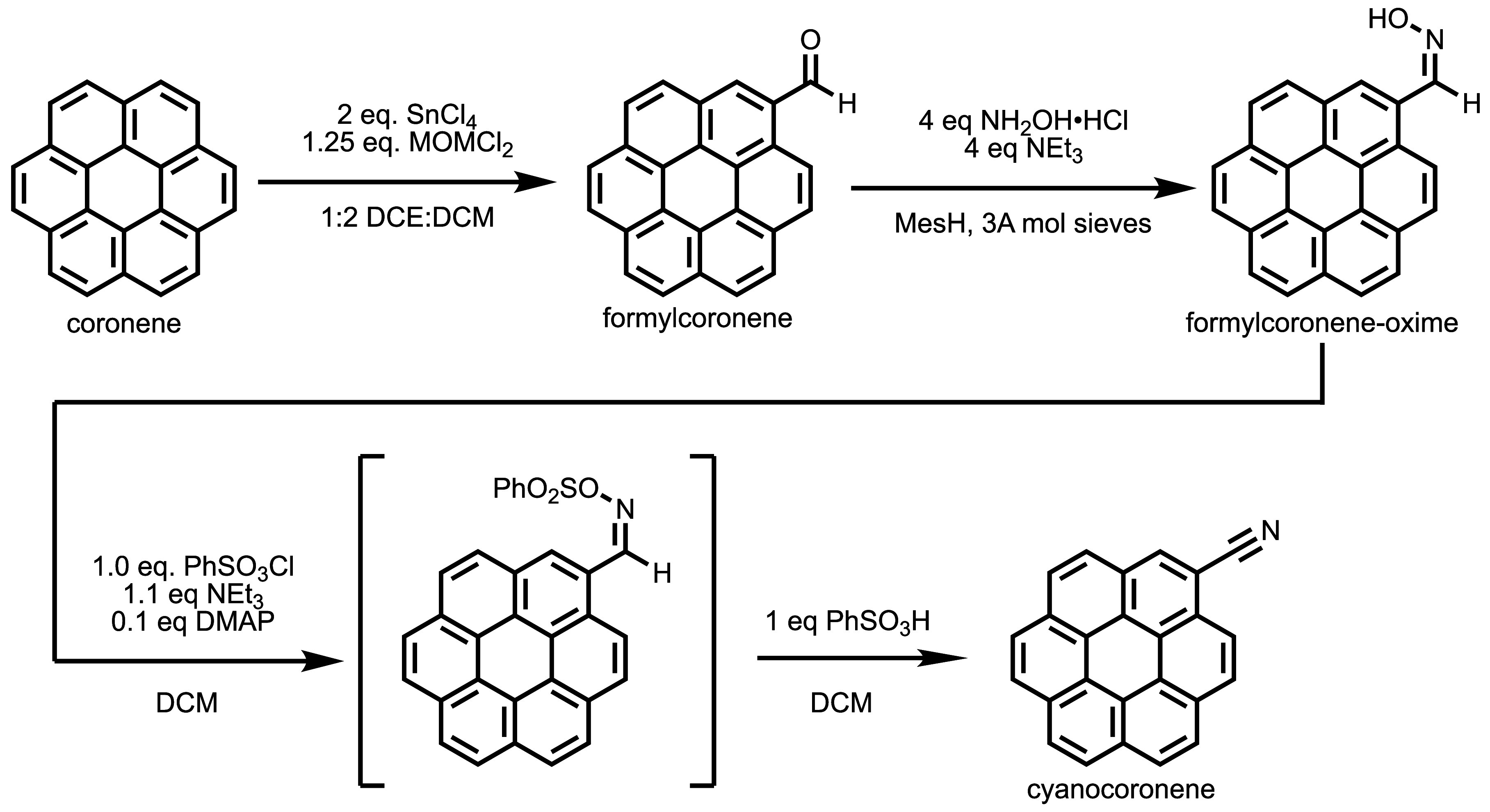
Full synthesis route from coronene (C_24_H_12_) to cyanocoronene (C_24_H_11_CN).

**Figure A2.**
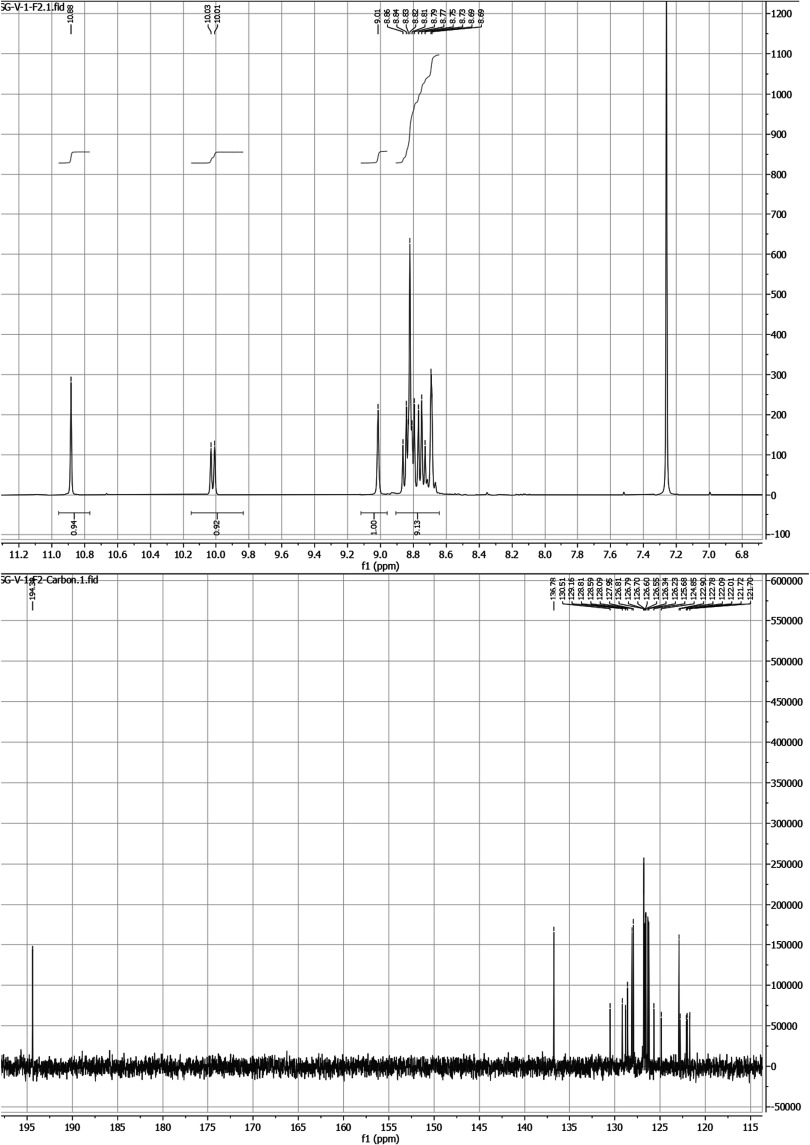
^1^H NMR (top) and ^13^C NMR (bottom) spectrum of formylcoronene.

**Figure A3.**
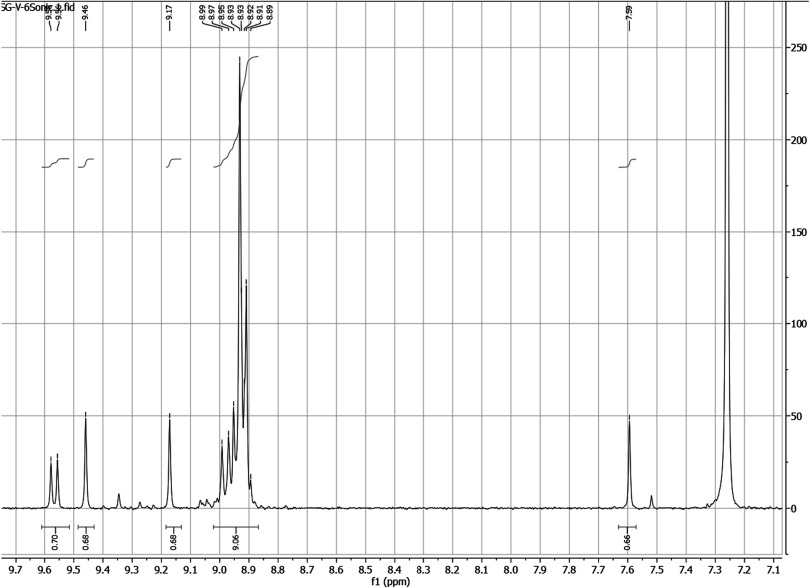
^1^H NMR spectrum of formylcoronene-oxime.

**Figure A4.**
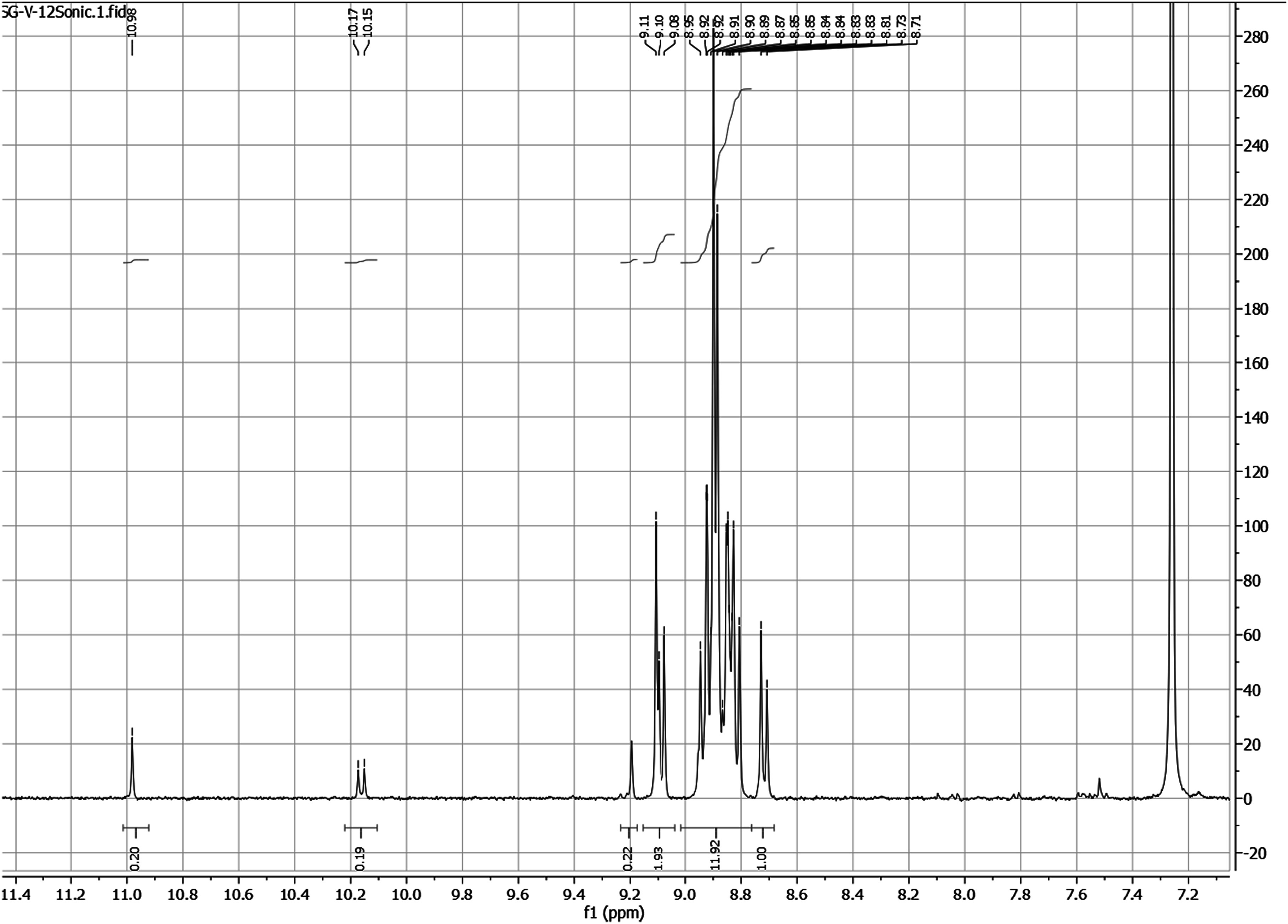
^1^H NMR spectrum of cyanocoronene.

**Figure C1.**
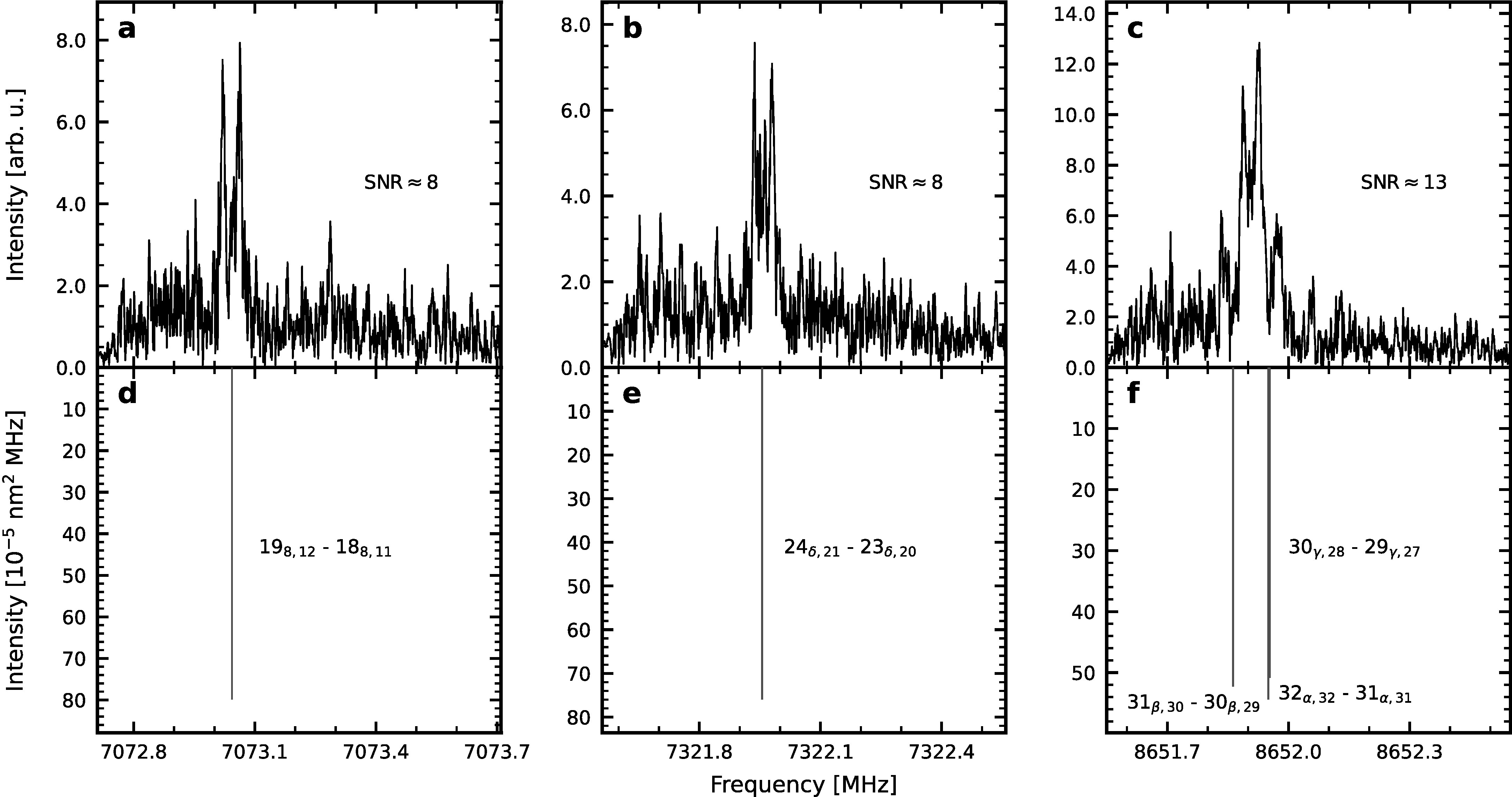
Example transitions of cyanocoronene recorded with the cavity-enhanced FTMW spectrometer. (a)–(c) Three example spectra that were recorded by averaging approximately 4500–10,000 gas pulses acquired at a 5 Hz repetition rate and a 1 *μ*s excitation pulse. The lines are split due to Doppler doubling induced by the coaxial cavity–supersonic expansion geometry of the instrument. We also report the SNR of the recorded lines. (d)–(f) Orange lines indicate the fitted rest frequencies. Corresponding quantum numbers of the transitions, ${J}_{{K}_{a}^{{\prime} },{K}_{c}^{{\prime} }}^{{\prime} }-J{{\prime\prime} }_{{K}_{a}^{\prime\prime} ,{K}_{c}^{\prime\prime} }$, are labeled. Closely overlapping *K*_*a*_ components are labeled with *α* = {0, 1}, *β* = {1, 2}, *γ* = {2, 3}, *δ* = {3, 4}.

**Figure E1.**
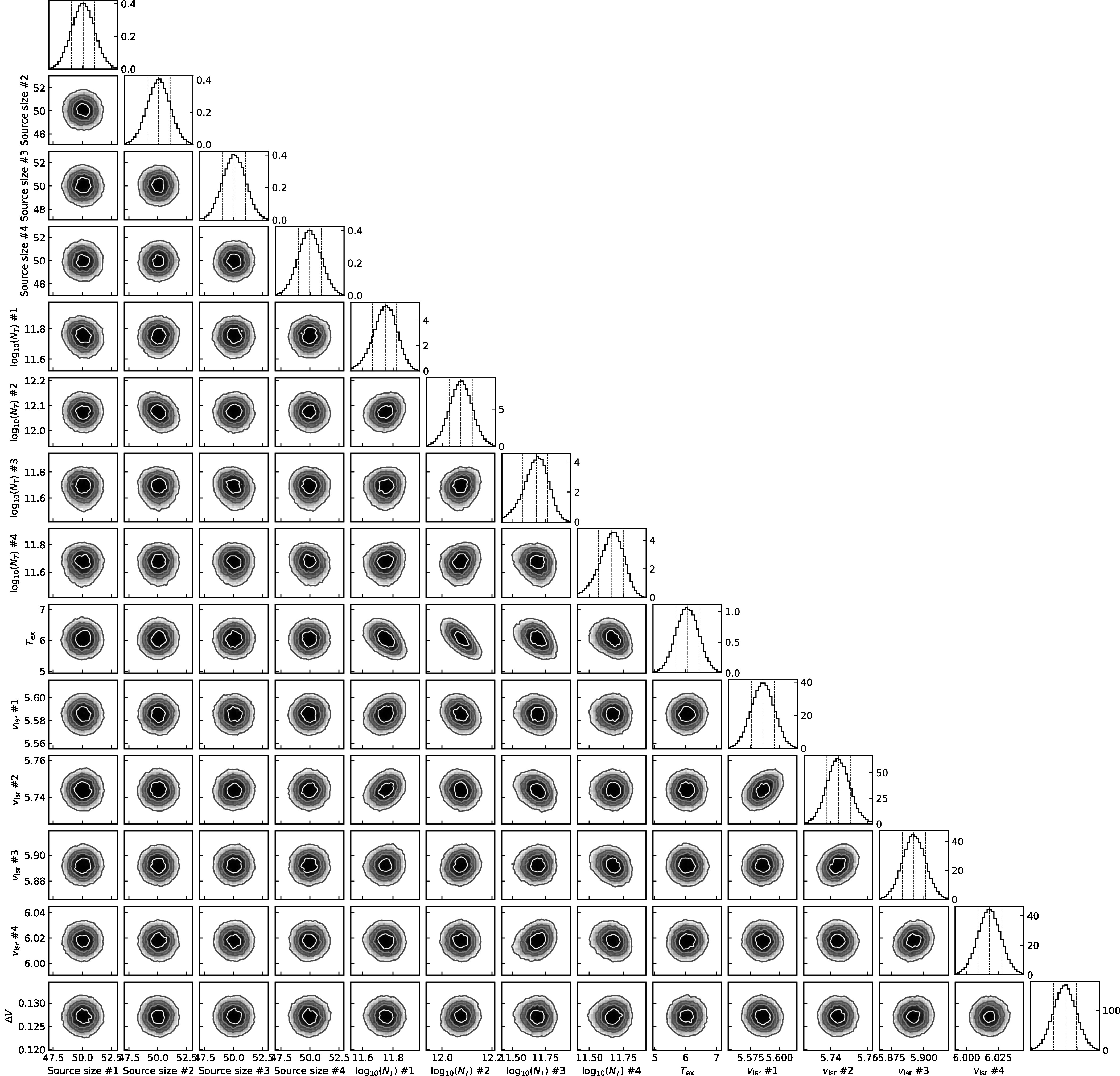
Parameter covariances and marginalized posterior distributions for the MCMC fit of cyanocoronene. The 16th, 50th, and 84th confidence intervals (corresponding to ±1*σ* for a Gaussian posterior distribution) are depicted as vertical lines on the diagonal plots.

**Figure G1.**
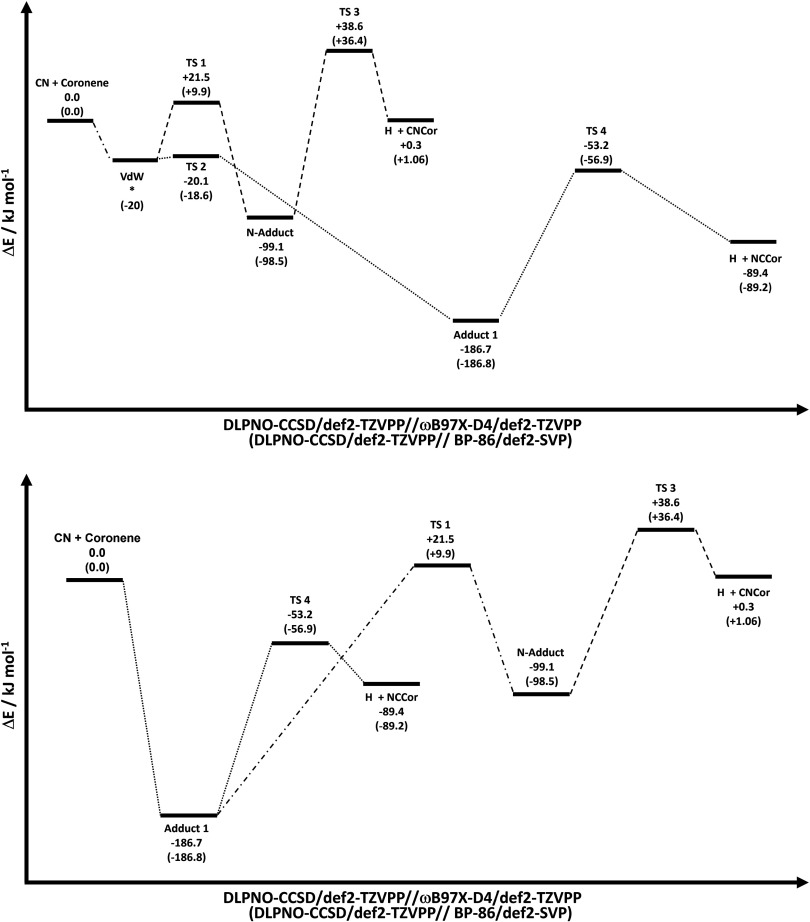
Potential energy surfaces for the addition of CN to coronene at the DLPNO-CCSD/def2-TZVPP//*ω*B97X-D4/def2-TZVPP level with DLPNO-CCSD/def2-TZVPP//BP-86-D3(BJ)/def2-SVP level results presented in parentheses. The upper panel provides the surface where a loose entrance channel complex is linked to the formation of the adducts. In the lower panel, this complex is neglected with a barrierless approach forming the cyano-adduct.

**Figure G2.**
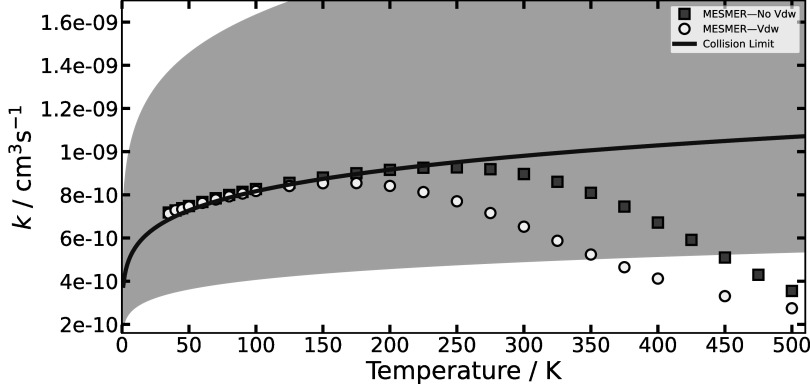
MESMER-simulated bimolecular rate coefficients leading to the formation of H and cyanocoronene from the reaction of CN with coronene with the CCT collision rate coefficient given as a solid blue curve; the estimated uncertainty in this is presented as the shaded region. The MESMER predictions including the loose complex on the entrance channel are given as yellow circles, and those neglecting this are given as red squares.

## Appendix A Full Synthesis Route to Cyanocoronene

The full synthesis route from coronene to cyanocoronene is depicted in Figure A1 and described in the following sections.

### A.1. Synthesis of Formylcoronene

Formylcoronene was prepared in accordance with the literature (T. J. Dale & J. Rebek 2006). To an oven-dried 500 mL Schlenk flask was added coronene (Ambeed Inc., 5.00 g, 16.7 mmol, 1 eq). After purging with N_2_, 80 mL anhydrous 1,2-dichloroethane (DCE) and 160 mL anhydrous dichloromethane (DCM) were added. The flask was then chilled to 0°C in an ice bath, and anhydrous SnCl_4_ (3.9 mL, 33 mmol, 2 eq) was injected into the mixture. Over the course of an hour, dichloromethyl methyl ether (MOMCl_2_, 1.9 mL, 21 mmol, 1.25 eq) was dropwise added into the mixture while the temperature was maintained at 0°C. The reaction was stirred for an additional 2 hr at 0°C before warming to 55°C and held at that temperature for 30 minutes. It was subsequently gradually cooled to room temperature and stirred for 19 hr. The reaction mixture was again cooled to 0°C, and 100 mL ice-cold water was added. After stirring for 3 hr at room temperature, the reaction was extracted five times with 100 mL DCM each. Anhydrous Na_2_SO_4_ was added to the organic phase, and the whole crude mixture was subjected to column chromatography using pure DCM to obtain formylcoronene (2.9 g, 8.8 mmol, 53% yield) as yellow needle-like crystals. ^1^H nuclear magnetic resonance (NMR; 400 MHz, CDCl_3_): *δ* 10.88 (s, 1H), 10.02 (d, *J* = 8.8 Hz, 1H), 9.01 (s, 1H), 8.91—8.64 (m, 9H). ^13^C NMR (126 MHz, CDCl_3_) *δ* 194.39, 136.78, 130.51, 129.16, 128.81, 128.59, 128.09, 127.95, 126.81, 126.79, 126.70, 126.60, 126.55, 126.34, 126.23, 125.68, 124.85, 122.90, 122.78, 122.09, 122.01, 121.72, 121.70 (peaks may overlap). Both are depicted in Figure A2.

We provide a list of potential difficulties that may be encountered during the synthesis of the target material and give guidance how to troubleshoot them:

1. The reaction will release HCl gas during heating. Caution is advised.
2. The reaction did not show improvement in yield after doubling the equivalence of SnCl_4_ and MOMCl_2_. However, most of the starting material is recoverable.
3. The product and unreacted starting material are both poorly soluble in a wide range of organic solvents. As such, copious amounts (potentially exceeding 6 L) of DCM are required to perform chromatography at the scale indicated above. It is also recommended that the chromatography is completed as quickly as possible since crystallized products can severely clog the column.

### A.2. Synthesis of Formylcoronene-oxime

To a 350 mL pressure vessel was added formylcoronene (1.31 g, 4.00 mmol, 1 eq), hydroxylammonium chloride (1.12 g, 16.0 mmol, 4 eq), powdered 3 Å mol sieves (800 mg), mesitylene (40 mL) and triethylamine (2.2 mL, 16 mmol, 4 eq) in the above order. The vessel was immediately sealed and heated at 150°C overnight. The mixture was then diluted with 500 mL tetrahydrofuran (THF) and filtered. The THF was removed via rotary evaporator, and 100 mL hexanes were added to precipitate the remaining formylcoronene-oxime. The solid precipitate was then filtered and washed with hexanes and methanol. The resulting solid was dried under vacuum to give formylcoronene-oxime (1.24 g, 3.61 mmol, 90% yield) as a brown granular powder. ^1^H NMR (400 MHz, CDCl_3_): *δ* 9.57 (d, *J* = 8.9 Hz, 1H), 9.46 (s, 1H), 9.17 (s, 1H), 9.02—8.87 (m, 9H), 7.59 (s, 1H), shown in Figure A3.

Troubleshooting:

1. The reaction mixture is heterogeneous and can stick on the walls of the vessel if stirred too violently, thus leading to incomplete reaction. Slow stirring is preferred.

### A.3. Synthesis of Cyanocoronene

Cyanocoronene was prepared according to a modified literature procedure (K. Hyodo et al. 2017): To an oven-dried round bottom flask under N_2_ was added formylcoronene-oxime (1.37 g, 4 mmol), 4-dimethylaminopyridine (DMAP; 49 mg, 0.4 mmol), DCM (20 mL), triethylamine (NEt_3_, 0.61 mL, 4.4 mmol) and PhSO3Cl (0.51 mL, 4 mmol). The reaction was stirred overnight. Once the oxime was fully consumed via ^1^H NMR analysis, PhSO3H (632 mg, 4 mmol) was dissolved in DCM (4 mL) and injected into the reaction mixture. After stirring overnight, the reaction was quenched with NEt_3_ and filtered through a silica pad with DCM as the eluent to obtain the product mixed with around 20% co-eluting formylcoronene. The solvents were removed under reduced pressure to obtain a yellow solid (920 mg). From the ratio of formylcoronene and cyanocoronene, the yield of cyanocoronene was calculated to be 2.35 mmol (59%). ^1^H NMR of the mixture (400 MHz, CDCl_3_): *δ* 10.98 (s, 0.2H, formylcoronene), 10.16 (d, *J* = 8.8 Hz, 0.2H, formylcoronene), 9.20 (s, 0.2H, formylcoronene), 9.13—9.05 (m, 2H, cyanocoronene), 8.98—8.79 (m, 12H, overlap), 8.72 (d, *J* = 8.5 Hz, 1H, cyanocoronene), depicted in Figure A4.

Troubleshooting:

1. Direct conversion of formylcoronene to cyanocoronene according to literature protocol (K. Hyodo et al. 2017) is difficult due to the limited reactivity of formylcoronene at room temperature. Noticeable conversion is only reliably achieved in refluxing toluene, chlorobenzene, or mesitylene. However, the acidic reaction conditions combined with high temperatures frequently resulted in significant degradation of the starting material and severely decreased yields.
2. The O-sulfonyl oxime intermediate is sensitive to hydrolysis. Its isolation is not recommended.
3. The reaction mixture is heterogeneous and can stick on the walls of the vessel if stirred too violently, thus leading to incomplete reaction. Slow stirring is preferred.
4. Copious amounts (∼8 L of DCM) were required to complete the chromatography due to the low solubility of the product. Precipitation-induced clogging can be mitigated by gently disturbing the top of the column.

## Appendix B Benchmark of Level of Theory

To benchmark the level of theory employed in this work, B3LYP/aug-cc-pVTZ, we provide in Table B1 theoretically computed rotational constants and quartic centrifugal distortion constants derived from the harmonic force field for the three cyanopyrene isomers that we previously studied using laboratory rotational spectroscopy (G. Wenzel et al. 2024, 2025). The MAPE comparing theoretical to experimental values for all three cyanopyrene isomers was 0.48%, and we therefore concluded that the B3LYP/aug-cc-pVTZ combination represents well the spectroscopic constants calculated for PAHs of this size.

**Table B1:** Rotational Constants of Three Cyanopyrene Isomers in the A-reduced I^*r*^ Representation

	1-cyanopyrene		
Parameter	Theoretical	Experimental^a^^,^^b^	Theoretical+Experimental^a^^,^^c^
*A* (MHz)	850.141	843.140191(128)	843.141827(128)
*B* (MHz)	372.931	372.500175(56)	372.500183(56)
*C* (MHz)	259.219	258.4249175(164)	258.4248913(164)
Δ_*J*_ (Hz)	1.977	2.240(80)	2.153(80))
Δ_*JK*_ (Hz)	−5.556	−5.52(101)	−5.88(101)
Δ_*K*_ (Hz)	20.310	[0]	[20.310]
*δ*_*J*_ (Hz)	0.724	0.826(40)	0.795(40)
*δ*_*K*_ (Hz)	3.059	5.26(74)	4.20(74)

	2-cyanopyrene		
Parameter	Theoretical	Experimental^a^^,^^b^	Theoretical+Experimental^a^^,^^c^
*A* (MHz)	1015.239	1009.19382(60)	1009.19356(39)
*B* (MHz)	314.479	313.1345299(202)	313.1345270(195)
*C* (MHz)	240.105	239.0427225(184)	239.0427270(167)
Δ_*J*_ (Hz)	0.706	0.7008(109)	0.7023(106)
Δ_*JK*_ (Hz)	5.375	5.814(94)	5.818(94)
Δ_*K*_ (Hz)	8.785	15.3(114)	[8.785]
*δ*_*J*_ (Hz)	0.184	0.1759(60)	0.1766(59)
*δ*_*K*_ (Hz)	4.510	4.22(39)	4.12(35)

	4-cyanopyrene		
Parameter	Theoretical	Experimental^a^^,^^b^	Theoretical+Experimental^a^^,^^c^
*A* (MHz)	652.155	651.383034(69)	651.382955(64)
*B* (MHz)	456.670	453.731352(45)	453.731444(41)
*C* (MHz)	268.590	267.5078168(224)	267.5078004(221)
Δ_*J*_ (Hz)	1.893	1.780(98)	2.026(69)
Δ_*JK*_ (Hz)	-1.553	0.43(58)	[-1.553]
Δ_*K*_ (Hz)	14.821	10.89(73)	12.61(53)
*δ*_*J*_ (Hz)	0.756	0.677(49)	0.803(35)
*δ*_*K*_ (Hz)	1.971	2.56(33)	2.40(33)

**Notes.** Experimental values were taken from G. Wenzel et al. (2024, 2025), theoretical values were calculated using the B3LYP/aug-cc-pVTZ level of theory, and the results of the combined theoretical and experimental fit are reported.

^a^ Values in parentheses are 1*σ* uncertainties in units of the last digit.

^b^ Values are from G. Wenzel et al. (2024, 2025). The value in brackets was not determinable and fixed to zero.

^c^ Values in brackets possessed large uncertainties and were fixed to their respective theoretical constants.

## Appendix C Measured Lines of Cyanocoronene

The experimentally measured lines of cyanocoronene are presented in Table C1. Examples of experimentally recorded transitions using the cavity-enhanced FTMW spectrometer are depicted in Figure C1.

**Table C1:** Experimentally Measured Transitions of Cyanocoronene between 6788 and 10520 MHz

Transition	Frequency^a^
(${J}_{{K}_{a}^{\prime} ,K{{\prime} }_{c}^{\prime} }-J{{\prime\prime} }_{{K}_{a}^{\prime\prime} ,{K}_{c}^{\prime\prime} }$)	(MHz)
24_1,23_–23_1,22_	6788.4098
24_2,23_–23_2,22_	6788.4098
25_0,25_–24_0,24_	6788.4443
25_1,25_–24_1,24_	6788.4443
23_2,21_–22_2,20_	6788.7145
23_3,21_–22_3,20_	6788.7145
22_4,19_–21_4,18_	6789.9187
22_3,19_–21_3,18_	6789.9187
19_7,13_–18_7,12_	6815.8858
18_7,11_–17_7,10_	7005.2518
25_1,24_–24_1,23_	7054.6106
25_2,24_–24_2,23_	7054.6106
26_0,26_–25_0,25_	7054.6617
26_1,26_–25_1,25_	7054.6617
24_2,22_–23_2,21_	7054.8759
24_3,22_–23_3,21_	7054.8759
23_3,20_–22_3,19_	7055.9209
23_4,20_–22_4,19_	7055.9209
22_5,18_–21_5,17_	7058.7857
22_4,18_–21_4,17_	7058.7857
19_8,12_–18_8,11_	7073.0418
20_7,14_–19_7,13_	7080.6353
20_6,14_–19_6,13_	7088.6004
18_8,10_–17_8,9_	7291.4710
26_1,25_–25_1,24_	7320.8179
26_2,25_–25_2,24_	7320.8179
27_0,27_–26_0,26_	7320.8780
27_1,27_–26_1,26_	7320.8780
25_2,23_–24_2,22_	7321.0420
25_3,23_–24_3,22_	7321.0420
24_3,21_–23_3,20_	7321.9574
24_4,21_–23_4,20_	7321.9574
23_5,19_–22_5,18_	7324.4393
23_4,19_–22_4,18_	7324.4393
27_1,26_–26_1,25_	7587.0283
27_2,26_–26_2,25_	7587.0283
28_0,28_–27_0,27_	7587.0875
28_1,28_–27_1,27_	7587.0875
22_10,13_–21_10,12_	8377.6038
30_1,29_–29_1,28_	8385.6505
30_2,29_–29_2,28_	8385.6505
31_0,31_–30_0,30_	8385.7347
31_1,31_–30_1,30_	8385.7347
29_2,27_–28_2,26_	8385.7636
29_3,27_–28_3,26_	8385.7636
26_6,21_–25_6,20_	8391.1806
26_5,21_–25_5,20_	8391.1806
31_1,30_–30_1,29_	8651.8594
31_2,30_–30_2,29_	8651.8594
32_0,32_–31_0,31_	8651.9466
32_1,32_–31_1,31_	8651.9466
30_2,28_–29_2,27_	8651.9537
30_3,28_–29_3,27_	8651.9537
29_3,26_–28_3,25_	8652.4539
29_4,26_–28_4,25_	8652.4539
28_4,24_–27_4,23_	8653.7843
28_5,24_–27_5,23_	8653.7843
27_6,22_–26_6,21_	8656.7527
27_5,22_–26_5,21_	8656.7527
37_2,35_–36_2,34_	10515.3437
37_3,35_–36_3,34_	10515.3437
38_1,37_–37_1,36_	10515.3437
38_2,37_–37_2,36_	10515.3437
39_0,39_–38_0,38_	10515.4529
39_1,39_–38_1,38_	10515.4529
35_4,31_–34_4,30_	10516.2356
35_5,31_–34_5,30_	10516.2356
34_5,29_–33_5,28_	10517.6703
34_6,29_–33_6,28_	10517.6703
33_6,27_–32_6,26_	10520.4313
33_7,27_–32_7,26_	10520.4313

**Note.**

^a^ Experimental uncertainties are 2 kHz.

## Appendix D Partition Function for Cyanocoronene

The partition function calculated by SPCAT for cyanocoronene, excluding ^14^N-quadrupole hyperfine splitting, is reported in Table D1.

**Table D1:** Partition Function for Cyanocoronene That Was Calculated by SPCAT for ${J}_{{\mathrm{\max }}}=400$ and ${K}_{{\mathrm{\max }}}=355$ and Used in the MCMC Analysis

Temperature	Partition Function
(K)	
1.0	1703.7230
2.0	4813.7245
3.0	8840.2413
4.0	13608.0333
5.0	19015.7897
6.0	24995.1437
7.0	31495.9011
8.0	38479.1687
9.375	48812.4929
18.75	138047.6140
37.5	390439.4924
75.0	1104317.3292
150.0	3120078.8657
225.0	5686759.1241
300.0	8589526.6725
400.0	12678742.2060
500.0	16744897.1786

## Appendix E MCMC Analysis of Cyanocoronene in TMC-1

The priors used for the MCMC analysis are reported in Table E1, and the resulting marginalized posterior distributions are listed and depicted in Table E2 and Figure E1, respectively. Note that we ran our MCMC analysis on 7768 transitions that have an uncertainty of lower than 5 kHz in the cyanocoronene catalog. The priors for velocity, *v*_LSR_; source size; and line width, Δ*V*, were heavily constrained with Gaussian distributions ${ \mathcal N }(\mu ,{\sigma }^{2})$ with mean, *μ*, and variance, *σ*^2^, informed from prior observations of PAHs (B. A. McGuire et al. 2021; G. Wenzel et al. 2024, 2025), while the column density, *N*_T_, and excitation temperature, *T*_ex_, were allowed to vary freely using a uniform (unweighted) distribution, ${ \mathcal U }\{a,b\}$, between *a* and *b*.

**Table E1:** Priors Used for the MCMC Analysis

Component	*v*_LSR_	Size	${{\mathrm{log}}}_{10}({N}_{{\mathrm{T}}})$	*T*_ex_	Δ*V*
No.	(km s^−1^)	(arcsec)	(cm^−2^)	(K)	(km s^−1^)
1	${ \mathcal N }(5.575,0.01)$				
2	${ \mathcal N }(5.767,0.01)$				
3	${ \mathcal N }(5.892,0.01)$	${ \mathcal N }(50,1)$	${ \mathcal U }\{{\mathrm{a}},{\mathrm{b}}\}$	${ \mathcal U }\{{\mathrm{a}},{\mathrm{b}}\}$	${ \mathcal N }(0.125,0.005)$
4	${ \mathcal N }(6.018,0.01)$				

Minimum	0.0	1	10.0	3.0	0.1
Maximum	10.0	100	13.0	15.0	0.3

**Note.** A Gaussian distribution, ${ \mathcal N }(\mu ,{\sigma }^{2})$, with mean *μ* and variance *σ*^2^, was used for the source size; velocity, *v*_LSR_; line width, Δ*V*; uniform (unweighted) distribution, ${ \mathcal U }\{a,b\}$, between *a* and *b* was used for the column density, *N*_T_; and excitation temperature, *T*_ex_.

**Table E2:** Summary Statistics of the Marginalized Posterior Probability Distributions from the MCMC Analysis for Cyanocoronene

Component	*v*_LSR_	Size	*N*_T_	*T*_ex_	Δ*V*
No.	(km s^−1^)	(arcsec)	(10^12^ cm^−2^)	(K)	(km s^−1^)
1	$5.58{6}_{-0.010}^{+0.010}$	50	$0.5{6}_{-0.10}^{+0.11}$		
2	$5.74{4}_{-0.006}^{+0.006}$	50	$1.1{9}_{-0.12}^{+0.13}$		
3	$5.89{2}_{-0.009}^{+0.009}$	50	$0.4{8}_{-0.10}^{+0.11}$	$6.0{5}_{-0.37}^{+0.38}$	$0.12{7}_{-0.002}^{+0.002}$
4	$6.01{8}_{-0.009}^{+0.009}$	49	$0.4{6}_{-0.09}^{+0.10}$		

${N}_{{\mathrm{T}}}({{\mathrm{C}}}_{24}{{\mathrm{H}}}_{11}{\mathrm{C}}{\mathrm{N}})={N}_{{\mathrm{T}}}({\mathrm{s}}{\mathrm{u}}{\mathrm{m}}{\mathrm{m}}{\mathrm{e}}{\mathrm{d}})=\,2.6{9}_{-0.23}^{+0.26}\times 1{0}^{12}\,{{\mathrm{c}}{\mathrm{m}}}^{-2}$					

**Note.** Priors used are reported in Table E1. The uncertainties are represented by the 16th and 84th percentiles, also known as the 68% confidence interval, which corresponds to 1*σ* for a Gaussian distribution. The total column density and its uncertainty are derived by marginalizing over the posterior distributions for the column densities of each individual component and reporting the 50th, 16th, and 84th percentiles. Source sizes were artificially overconstrained (see G. Wenzel et al. 2024), and hence, we do not report their uncertainties.

## Appendix F Column Densities of Cyclic Hydrocarbons in TMC-1

The data compiled in Figure 4 are listed in Table F1.

**Table F1:** Column Densities of Carbon-bearing Cycles Observed in TMC-1 as Presented in Figure 4

Molecule	*N*_C_	Column Density	Reference
	(10^12^ cm^−2^)		
C_5_H_6_	5	12${}_{-3}^{+3}$	J. Cernicharo et al. (2021a)
C_6_H_4_	6	0.5${}_{-0.1}^{+0.1}$	J. Cernicharo et al. (2021b)
1-C_5_H_5_CN	6	0.827${}_{-0.100}^{+0.090}$	K. L. K. Lee et al. (2021)
2-C_5_H_5_CN	6	0.189${}_{-0.015}^{+0.018}$	K. L. K. Lee et al. (2021)
1-C_5_H_5_CCH	7	1.4${}_{-0.2}^{+0.2}$	J. Cernicharo et al. (2021c)
2-C_5_H_5_CCH	7	2.0${}_{-0.4}^{+0.4}$	J. Cernicharo et al. (2021c)
C_5_H_4_CCH_2_	7	2.7${}_{-0.3}^{+0.3}$	J. Cernicharo et al. (2022)
C_6_H_5_CN	7	1.73${}_{-0.10}^{+0.85}$	B. A. McGuire et al. (2021)
C_6_H_5_CCH	8	3.0${}_{-0.5}^{+0.5}$	D. Loru et al. (2023)
C_9_H_8_	9	9.04${}_{-0.96}^{+0.96}$	M. L. Sita et al. (2022)
2-C_9_H_7_CN	10	0.210${}_{-0.046}^{+0.060}$	M. L. Sita et al. (2022)
1-C_10_H_7_CN	11	0.74${}_{-0.46}^{+0.33}$	B. A. McGuire et al. (2021)
2-C_10_H_7_CN	11	0.71${}_{-0.32}^{+0.45}$	B. A. McGuire et al. (2021)
1-C_12_H_7_CN	13	0.95${}_{-0.09}^{+0.09}$	J. Cernicharo et al. (2024)
5-C_12_H_7_CN	13	0.95${}_{-0.09}^{+0.09}$	J. Cernicharo et al. (2024)
1-C_16_H_9_CN	17	1.52${}_{-0.16}^{+0.18}$	G. Wenzel et al. (2024)
2-C_16_H_9_CN	17	0.84${}_{-0.09}^{+0.09}$	G. Wenzel et al. (2025)
4-C_16_H_9_CN	17	1.33${}_{-0.09}^{+0.10}$	G. Wenzel et al. (2025)
C_16_H_10_^a^	16	∼30	G. Wenzel et al. (2025)
C_24_H_11_CN	25	2.69${}_{-0.23}^{+0.26}$	This work
C_24_H_12_^a^	24	∼20	This work

**Note.**

^a^ Estimated using the cyanopyrene and cyanocoronene column densities, calculated rate coefficients for CN addition, and a CN/H ratio of 0.15 (see Appendix G and G. Wenzel et al. 2025).

## Appendix G Ab Initio Quantum Chemical Calculations

Master equation calculations were performed in MESMER 7.1 (D. R. Glowacki et al. 2012) on an ab initio surface calculated at the DLPNO-CCSD/def2-TZVPP//*ω*B97X-D4/def2-TZVPP level (F. Weigend 2006; J.-D. Chai & M. Head-Gordon 2008; E. Caldeweyher et al. 2017) in ORCA 5.0.4 (F. Neese 2022); the master equation calculations showed that below 100 K with a gas density of 2 × 10^4^ cm^−3^, the reaction of CN with coronene leads to the formation of cyanocoronene and an H atom at the collision rate. The collision rate is predicted to be ${k}_{{\mathrm{col}}}\,=5.{6}_{-2.8}^{+5.6}\times 1{0}^{-10}\,{{\mathrm{cm}}}^{3}\,{{\mathrm{s}}}^{-1}$ using classical capture theory in the manner described in N. A. West et al. (2019) and G. Wenzel et al. (2025), with no prediction of the formation of isocyanocoronene. Using this collision rate and its relationship to the CN/H ratio on formation rate from G. Wenzel et al. (2025), CN/H = 0.15, leads to a ratio of coronene to cyanocoronene of 6.67 and a column density of coronene of *N*(C_24_H_12_) ≈ 2 × 10^13^ cm^−2^.

### G.1. Computational Methodology

Calculations of a potential energy surface for the formation of cyanocoronene were carried out in ORCA 5.0.4 (F. Neese 2022). Initial structures for the adducts and separated reagents were optimized with the RI-BP86 DFT functional (J. P. Perdew 1986; A. D. Becke 1988; C. Lee et al. 1988) using the def2-SVP basis set (F. Weigend & R. Ahlrichs 2005) and including D3(BJ) empirical dispersion corrections (A. D. Becke & E. R. Johnson 2006; S. Grimme et al. 2011; this method will be subsequently referred to as DFT-Cheap). The presence of barriers to the addition of CN to coronene and the subsequent elimination of a H atom were evaluated by carrying out modified redundant scans of the forming or breaking bonds. In these scans, all coordinates except the bond being scanned were allowed to relax while the scanned coordinate was varied. The resultant maxima were used as input geometries for transition-state optimizations, and the long-range minima prior to CN addition were optimized to a loose bound complex on the entrance channel.

Harmonic vibrational analysis was carried out to verify that these structures were indeed stationary points, and the resulting frequencies were scaled by 0.9956 and harmonic zero-point energies were scaled 1.0207 in the manner suggested by M. K. Kesharwani et al. (2015). Intrinsic reaction coordinate scans were performed to verify that transition states linked reactants and products (K. Ishida et al. 1977; F. Neese 2022). The structures found by this approach were re-optimized with the hybrid *ω*B97X functional (J.-D. Chai & M. Head-Gordon 2008) with the D4 empirical dispersion correction (E. Caldeweyher et al. 2017) and the def2-TZVPP triple zeta basis set (F. Weigend 2006; this method will subsequently be referred to as DFT-2). Harmonic vibrational frequencies were calculated on the DFT-2 structures, and the results were scaled by 0.9533 and harmonic zero-point energies were scaled 0.9779 in the manner suggested by M. K. Kesharwani et al. (2015).

Single-point energy corrections were carried out using the domain pair local natural orbital coupled cluster method including single and double excitations DLPNO-CCSD, with tight thresholds for the pair natural orbitals (PNOs; P. Pinski et al. 2015; C. Riplinger et al. 2016). This method was used with two basis sets, first, using Ahlrichs triple zeta def2-TZVPP basis set and second with Dunning’s augmented double-zeta correlation consistent basis set aug-cc-pVDZ (T. H. Dunning 1989; R. A. Kendall et al. 1992; E. R. Davidson 1996). These single-point energy corrections were calculated for both of the structures calculated with DFT-Cheap and DFT-2. For the reactants and products, additional single-point energy corrections were carried out using the EP3 scheme for approximating the complete basis set (CBS) CCSD(T) result. In the EP3 scheme a single CCSD(T)/cc-pVDZ calculation is combined with three MP2 calculations using three of Dunning’s correlation consistent basis set series cc-pVDZ, cc-pVTZ, and cc-pVQZ (T. H. Dunning 1989; E. R. Davidson 1996), taken from the correlation consistent basis set repository, ccREPO (G. Hill 2016). The Hartree-Fock energies (E_HF_) and MP2 correlation energies (E_MP2_) were extrapolated to the CBS limit and combined with the CCSD(T) correlation energy (E_CCSD(T)_) in Equation (G1) to calculate an EP3 approximation of the CCSD(T) complete basis set limit (P. Jurečka et al. 2006; D. G. Liakos & F. Neese 2012):

\begin{eqnarray*}\begin{array}{rcl}{\mathrm{CCSD(T)/CBS}} &amp; \approx &amp; {\mathrm{EP3}}={E}_{{\mathrm{HFCBS}}}+{E}_{{\mathrm{MP2CBS}}}\\ &amp; &amp; +{E}_{{\mathrm{CCSD(T)small}}}-{E}_{{\mathrm{MP2small}}}.\end{array}\end{eqnarray*}

A loose Van der Waals (VdW) complex was found using DFT-Cheap, which failed to re-optimize with DFT-2. Additional scans were carried out of the entrance channel with DFT-2, and although a shallow minima and submerged barrier were apparent on these, the shallow minima again did not optimize to loose complexes. Therefore, there is uncertainty in the presence of a loose entrance channel complex, and as such, two separate surfaces were treated in subsequent master equation calculations, one where both adducts were linked to an entrance channel complex with energetics defined at the DLPNO-CCSD//DFT-Cheap level and the second where no entrance channel barriers were present for the formation of the cyanocoronene adducts with all energetics at the DLPNO-CCSD//DFT-2 level. These surfaces are presented in Figure G1 and summarized in Table G1.

**Table G1:** Relative Energies for Selected Stationary Points on the CN + Coronene Potential Energy Surface Including Scaled Harmonic Zero-point Energies

	1-1	1-2	1-3	1-4	1-5	2-1	2-2	2-3	2-4	2-5
CN + Coronene	0.0	0.00	0.0	0.0	0.0	0.0	0.00	0.0	0.0	0.0
VdW	−20.57	−20.26	−25.82	⋯	⋯	–	–	–	⋯	⋯
TS 1 (CN addition)	9.87	14.42	3.83	⋯	⋯	21.48	25.03	13.18	⋯	⋯
N-Adduct 1	−98.48	−90.90	−98.57	⋯	⋯	−99.08	−91.60	−101.41	⋯	⋯
TS 3 H elimination	36.35	42.57	33.38	⋯	⋯	38.55	45.37	34.23	⋯	⋯
H + Isocyanocoronene	1.06	0.14	1.72	1.12	−16.20	0.33	−0.68	0.20	0.94	−14.17
TS 2 (NC addition)	−18.57	−18.27	−23.82	⋯	⋯	−20.09	−18.52	−25.05	⋯	⋯
Adduct 1	−186.80	−179.23	−184.50	⋯	⋯	−186.68	−179.22	−186.00	⋯	⋯
TS 4 H elimination	−56.87	−50.57	−57.91	⋯	⋯	−53.20	−46.27	−54.98	⋯	⋯
H + Cyanocoronene	−89.15	−89.97	−86.33	−89.88	−106.47	−89.39	−90.31	−86.53	−88.90	−103.67

**Note.** In the column headers, 1- represents using BP-86-D3(BJ)/def2-SVP structures and 2- represents using *ω*B97X-D4/def2-TZVPP structures, with the single-point energy corrections applied being given as -1 for DLPNO-CCSD/def2-TZVPP, -2 for ROHF-DLPNO-CCSD/def2-TZVPP -3 for DLPNO-CCSD/aug-cc-pVDZ, -4 for CCSD(T)/cc-pVDZ and -5 for EP3-MP2 (DZ-QZ). All values are reported in kJ mol^−1^. The "–" indicates that this approach was not successful and hence we cannot report values.

The relative energies given in Table G1 for both the DFT-Cheap and DFT-2 surfaces are in very good agreement for the overall reaction energetics once DLPNO-CCSD corrections have been applied both with the aug-cc-pVDZ and def2-TZVPP basis sets. In addition, the relative energies of the cyano and isocyano adducts formed by addition of CN to coronene and their subsequent barriers to elimination of a H atom are in excellent agreement (deviating by less than 1 kcal). Comparing to canonical couple cluster results including single and double excitations and perturbative triples with the cc-pVDZ basis set, the agreement for the overall energetics of the reaction are well reproduced by the DLPNO-CCSD results. Incorporation of basis set extrapolation through the EP3 scheme leads to a large deviation of around 15 kJ mol^−1^. Considering the whole set of results provided in Table G1, it is reasonable to infer that the formation of isocyanocoronene is slightly endothermic at 0 K. The effect of spin contamination was considered by carrying out further DLPNO-CCSD/def2-TZVPP calculations with spin restricted open shell Hartree–Fock (ROH-DLPNO-CCSD), as well as the spin-unrestricted (DLPNO-CCSD) calculations with the def2-TZVPP basis set. These results typically deviated by less than 10 kJ mol^−1^.

For both the UHF-DLPNO-CCSD//DFT-Cheap and UHF-DLPNO-CCSD//DFT-2 surfaces, emerged barriers were found for both the formation of the isocyanocoronene adduct (+9.9 and 21.5 kJ mol^−1^) and the subsequent H atom elimination, leading to the formation of isocyanocoronene (+36.4 and +38.6 kJ mol^−1^), whereas the formation of the cyanocoronene occurs over a submerged barrier in the entrance channel (−18.6 and −20.1 kJ mol^−1^) and the elimination of a H atom leading to the formation of cyanocoronene occurs over a very submerged barrier on the exit channel (−56.9 and −53.2 kJ mol^−1^).

### G.2. Master Equation Kinetic Predictions

The UHF-DLPNO-CCSD//DFT-2 surface including and excluding the UHF-DLPNO-CCSD//DFT-Cheap loose entrance channel complex was incorporated into the energy-grained master equation calculator MESMER 7.1 (D. R. Glowacki et al. 2012), which allowed the reaction to be simulated over a range of densities (1 × 10^4^–1 × 10^15^ cm^−3^) and temperatures (20–3000 K). Where the entrance channel complex was included in MESMER, simulations were primarily carried out with the UHF-DLPNO-CCSD-corrected DFT-Cheap barriers to addition. However, repeats were carried out with the UHF-DLPNO-CCSD//DFT-2 barrier to the formation of the isocyano-adduct (the UHF-DLPNO-CCSD//DFT-2 barrier to the formation of the cyano-adduct was submerged with respect to the UHF-DLPNO-CCSD//DFT-Cheap entrance channel complex). In addition, simulations were carried out where all the barriers were raised by 10 kJ mol^−1^ and where all the barriers were reduced by 10 kJ mol^−1^.

The temperature-dependent collision rate coefficient for the barrierless formation of the cyano-adduct or the loose complex was estimated using classical capture theory (CCT; Y. Georgievskii & S. J. Klippenstein 2005):

\begin{eqnarray*}\begin{array}{rcl}{k}_{\mathrm{coll}}(T) &amp; = &amp; {\sigma }_{\mathrm{coll}}\langle v(T)\rangle \\ &amp; = &amp; \left[\pi {\left(\displaystyle \frac{2{C}_{6}}{{k}_{{\mathrm{B}}}T}\right)}^{1/3}{\mathrm{\Gamma }}\left(\displaystyle \frac{2}{3}\right)\right]\left[{\left(\displaystyle \frac{8{k}_{{\mathrm{B}}}T}{\pi \mu }\right)}^{1/2}\right].\end{array}\end{eqnarray*}

In Equation (G2), *k*_B_ is the Boltzmann constant, Γ(*x*) is the gamma function such that Γ(2/3) = 1.353, *μ* is the reduced mass of the collision, and *C*_6_ is the sum of coefficients describing the magnitude of the attractive forces between collision partners given as Equation (G3):

\begin{eqnarray*}\begin{array}{rcl}{C}_{6} &amp; = &amp; \displaystyle \frac{2}{3}\left(\displaystyle \frac{{\mu }_{1}^{2}{\mu }_{2}^{2}}{{k}_{{\mathrm{B}}}T{\left(4\pi {\epsilon }_{0}\right)}^{2}}\right)+\displaystyle \frac{{\mu }_{1}^{2}{\alpha }_{2}+{\mu }_{2}^{2}{\alpha }_{1}}{4\pi {\epsilon }_{0}}\\ &amp; &amp; +\displaystyle \frac{3}{2}{\alpha }_{1}{\alpha }_{2}\left(\displaystyle \frac{{I}_{1}{I}_{2}}{{I}_{1}+{I}_{2}}\right).\end{array}\end{eqnarray*}

In Equation (G3), *ϵ*_0_ represents the permittivity of free space, *μ*_1_ and *μ*_2_ are the dipole moments of the reactants, and *α*_1_/*α*_2_ and *I*_1_/*I*_2_ are their polarizabilities and ionization energies, respectively. These parameters were taken from the online databases NIST Chemistry WebBook and CCCBDB (R. I. Johnson 2022; P. Linstrom & W. Mallard 2024). This approach has been shown to be accurate within a factor of 2 for the prediction of rate coefficients for barrierless radical neutral reactions (N. A. West et al. 2019). The redissociation of the adducts was then treated with the inverse Laplace transformation methodology (J. W. Davies et al. 1986) in MESMER. The subsequent reactions through defined transition states were treated with Rice–Ramsperger–Kassel–Marcus theory (T. Baer & W. L. Hase 1996; K. A. Holbrook et al. 1996; U. Lourderaj & W. L. Hase 2009) including quantum mechanical tunneling effects through a one-dimensional asymmetric Eckart barrier (W. H. Miller 1979).

Below 200 K, the results became sensitive to the grain size chosen, with a grain size of 10 cm^−1^ used for calculations under 200 K; even with a grain size of 2–5 cm^−1^, calculations failed below 20 K. From 125 to 20 K with a gas density of 2 × 10^4^ cm^−3^ in the complex included and from 200 K in the complex excluded case, the calculated bimolecular rate coefficients leading to the formation of cyanocoronene and a H atom from the reaction of CN and coronene matched the estimated collision rate as can be seen in Figure G2. Therefore, it is reasonable to predict that at a gas density of 2 × 10^4^ cm^−3^ and at 10 K, this reaction will occur at the collision rate (${k}_{{\mathrm{col}}}=5.{6}_{-2.8}^{+5.6}\times 1{0}^{-10}\,{{\mathrm{cm}}}^{3}\,{{\mathrm{s}}}^{-1}$) and lead solely to the formation of cyanocoronene (isocyanocoronene is not predicted to be formed under these conditions). In these simulations, the temperature-dependent energy transfer parameter <Δ*E*>_*d*_ was set as 80 × (T/298)^1.0^ cm^−1^ using the value assumed in Y. Georgievskii & S. J. Klippenstein (2005); additionally, it should be noted the result that the collision limit had been reached below 100 K was not sensitive to this parameter when it was varied between 50 and 150 × (T/298)^1.0^ cm^−1^.

As the route to the formation of isocyanocoronene is endothermic and involves emerged barriers, both in the entrance channel and to the elimination of an H atom leading to product formation, this channel is predicted to be negligible. Varying the height of these barriers by 10 kJ mol^−1^ did not result in any observed isocyanocorone formation. Repeats were additionally performed where these barriers were shifted to match the deviation between the DLPNO-CCSD/def2-TZVPP-corrected and EP3-MP2(DZ-QZ)-corrected energetics (≈15 kJ mol^−1^); the entrance channel barrier becomes submerged, but the exit barrier remains emerged, and the resulting prediction showed no formation of isocyanocoronene. The route to the formation of cyanocoronene involves barriers that are submerged both in the entrance channel and to the subsequent H atom elimination. The depth to which the barriers are submerged on the route to the formation of cyanocoronene means that this route remained rapid under the conditions relevant to TMC-1 even when the barriers were raised by 15 kJ mol^−1^.
